# Bacterial Surface Colonization of Sputter-Coated Platinum Films

**DOI:** 10.3390/ma13122674

**Published:** 2020-06-12

**Authors:** Dominika Czerwińska-Główka, Wioletta Przystaś, Ewa Zabłocka-Godlewska, Sebastian Student, Beata Cwalina, Mieczysław Łapkowski, Katarzyna Krukiewicz

**Affiliations:** 1 Department of Physical Chemistry and Technology of Polymers, Silesian University of Technology, 44-100 Gliwice, Poland; dominika.czerwinska-glowka@polsl.pl (D.C.-G.); mieczyslaw.lapkowski@polsl.pl (M.Ł.); 2 Department of Environmental Biotechnology, Faculty of Energy and Environmental Engineering, Silesian University of Technology, 44-100 Gliwice, Poland; wioletta.przystas@polsl.pl (W.P.); ewa.zablocka-godlewska@polsl.pl (E.Z.-G.); beata.cwalina@polsl.pl (B.C.); 3 Biotechnology Centre, Silesian University of Technology, 44-100 Gliwice, Poland; sebastian.student@polsl.pl; 4 Department of Systems Biology and Engineering, Faculty of Automatic Control, Electronics and Computer Science, Silesian University of Technology, 44-100 Gliwice, Poland

**Keywords:** antimicrobial properties, bacterial attachment, bacterial growth, *Escherichia coli*, glass, platinum, sputter-coating

## Abstract

Due to its biocompatibility and advantageous electrochemical properties, platinum is commonly used in the design of biomedical devices, e.g., surgical instruments, as well as electro-medical or orthopedic implants. This article verifies the hypothesis that a thin layer of sputter-coated platinum may possess antibacterial effects. The purpose of this research was to investigate the adhesion and growth ability of a model strain of Gram-negative bacteria, *Escherichia coli,* on a surface of a platinum-coated glass slide. Although some previous literature reports suggests that a thin layer of platinum would inhibit the formation of bacterial biofilm, the results of this study suggest otherwise. The decrease in the number of bacterial cells attached to the platinum-coated glass, which was observed within first three hours of culturing, was found to be a short-time effect, vanishing after 24 h. Consequently, it was shown that a thin layer of sputter-coated platinum did not exhibit any antibacterial effect. For this reason, this study indicates an urgent need for the development of new methods of surface modification that could reduce bacterial surface colonization of platinum-based biomedical devices.

## 1. Introduction

The antimicrobial properties of metals have been known for centuries [1]. Noble metal ions, particularly, have been indicated as potential antibacterial agents. For instance, Vaidya et al. [2] tested various solutions of metal ions (silver, copper, platinum, gold and palladium) for their antimicrobial properties against *Enterococcus faecium, Acinetobacter baumannii* and *Klebsiella pneumoniae,* confirming the superiority of platinum, gold and palladium over non-noble metal ions. Apart from antibacterial activity, noble metals, especially platinum, offer high strength and stability in different conditions, as well as biocompatibility. Furthermore, high electrical conductivity of platinum is an excellent property qualifying this metal for the design of pacemakers, hearing aids and neurological implants [3,4,5].

Antimicrobial effects may be also achieved by the use of metal nanoparticles. Consequently, nanoparticles of gold, silver, zinc, silica or platinum have been successfully used in the design of biomedical devices [6,7], as bactericidal [8,9,10,11] or antibiofouling agents [12,13]. For instance, Gopal et al. [14] studied the behavior of bacteria exposed to platinum nanoparticles with various sizes, showing the toxicity of small nanoparticles (1–3 nm) and bacterial tolerance of larger nanoparticles (4–21 nm). Small nanoparticles with a spherical morphology were supposed to pass through the pores of the bacterial membrane. In contrast, larger ones, possessing cuboid or flower shapes, were not able to penetrate the membrane. In other studies, Huang et al. [15] indicated a different mechanism of antibacterial action of silver nanoparticles coated with catechol-conjugated chitosan against Gram-negative (*Escherichia coli*) and Gram-positive (*Staphylococcus aureus*) bacteria. In the case of *Staphylococcus aureus*, nanoparticles induced damage to the cell wall and leakage of cytoplasmic proteins. In the case of *Escherichia coli,* nanoparticles were found to adsorb onto the surface of bacterial cells, leading to the change in permeability of a cell membrane, allowing the passage of silver ions into the cytoplasm and thus causing the damage to the bacterial cell. In the work of Kummala et al. [16], silver and titanium dioxide nanoparticles were deposited onto a glass surface by a liquid flame spray technique. This method allowed to distribute nanoparticles uniformly over the entire surface, with a layer thickness controlled through sputtering time. As-formed coatings exhibited antibacterial activity against Gram-positive (*Staphylococcus aureus*) and Gram-negative (*Escherichia coli*) bacteria, and were able to effectively reduce their growth.

Demonstration of antibacterial properties of noble metal ions, as well as their nanoparticles, suggested the desirability of investigating whether noble metals as coatings in a form of ultrathin layers would also exhibit antibacterial activity. Such a thin layer of metal (for example platinum) could be obtained by the use of sputtering techniques. Metal sputtering methods have been increasingly investigated as processes allowing to form a variety of unique materials. For instance, thin layers of copper and silver were sputtered alternately on glass and plastic surfaces, and their antibacterial activity was confirmed against *Staphylococcus epidermidis* and *Staphylococcus aureus* strains [17]. Similarly, Wang et al. [18] used radio frequency magnetron sputtering to deposit nanostructured silver films on the surface of polypropylene nonwovens. The antibacterial properties of as-formed layers were assessed against *Staphylococcus aureus* and *Escherichia coli*, demonstrating the enhancement of antibacterial effects with the thickness of silver layer. Furthermore, Musil [19] described flexible antibacterial coatings fabricated through a reactive magnetron sputtering method. Cr–Cu–O, Al–Cu–N and Zr–Cu–N coatings were given as examples, and their antibacterial properties were analyzed against *Escherichia coli*. It was shown that sputter-coating allowed to produce thick, durable and flexible coatings effective against model bacterial strain after just three hours of contact. Due to the fact that sputter-coating can be used to modify any type of surface, coatings obtained in this way have numerous potential industrial applications in a variety of fields, particularly in biomedical engineering.

In the light of previous research studies showing the antibacterial activity of surfaces modified through sputter-coating, it could be expected that also sputter-coated platinum films should possess similar biocidal characteristics. To verify whether a thin layer of platinum can serve as an efficient factor inhibiting the adhesion and multiplication of bacteria, we compared the behavior of *Escherichia coli* cultured on the surface of platinum-coated and noncoated (bare) glass slides. According to Gaynes et al. [20], *Escherichia coli* was the most commonly reported Gram-negative pathogen in 2013. In addition, *Escherichia coli* has been identified by Vihta et al. [21] as a major cause of bloodstream infection with a critical antimicrobial resistance. As bacterial culture was conducted in a closed system, *Escherichia coli* was expected to grow in a predictable way, giving a growth curve consisting of four different phases: the initial phase (lag phase) referring to metabolically active bacteria that may increase in cell mass, but not in the cell number; the logarithmic phase (log phase), in which a rapid exponential growth of bacteria population is observed; the stationary phase, in which the population of bacteria begins to decline as nutrients are consumed and inhibitory products are accumulated; and, finally, a death phase, in which bacterial cells die due to the lack of nutrients, the excess of toxic substances and also some harmful conditions [22,23,24]. Therefore, to extensively analyze the effect of surface modification on the attachment and growth of *Escherichia coli*, the bacterial cells were characterized in terms of their dimensions and vitality after 3 h, 24 h and 48 h, corresponding to the initial phase, logarithmic phase and death phase during the growth of bacteria. The biologic features were discussed together with the physicochemical characteristics of platinum and glass (roughness and hydrophilicity), revealing the relationship between the surface morphology of the materials and their interactions with microorganisms.

## 2. Materials and Methods

### 2.1. Preparation and Surface Characterization of Bare and Pt-Coated Glass Slides

A glass support substrate (microscopic slide, Labglass, 75 mm × 25 mm × 1 mm) was used as the reference material. To fabricate Pt-coated films, glass slides were sputter-coated with a thin layer of Pt (4.8 nm) using a sputter-coater (automatic, rotary-pump coating system, Q150R Quorum Technologies, Lewes, UK) working at 30 mA for 120 s. According to the specifications of the manufacturer, the conditions of the sputter-coating process allowed to produce a continuous platinum film with the grain size in the order of 2 nm. To compare both materials, their roughness was examined using scanning electron microscopy (Phenom ProX, Thermo Fisher Scientific, Waltham, MA, USA) and the 3D Roughness Reconstruction software (Phenom ProSuits, Thermo Fisher Scientific, Waltham, MA, USA). The wettability of materials was determined by contact angle measurements, by means of a DataPhysics OCA15 goniometer (DataPhysics Instruments, Filderstadt, Germany) using deionized water. In all measurements, three sample sites were analyzed on three different sample surfaces, giving n = 9.

### 2.2. Culturing Bacteria and Examining Their Growth on Bare and Pt-Coated Glass Slides

The bacterial strain of Gram-negative *Escherichia coli* (DSM 30083, U5/41) was cultured in 23 g/L agar broth (BTL, Warsaw, Poland) at 35 °C for 48 h in an incubator. After this time, the agar slants were washed with physiological salt (0.85% water solution of NaCl; Acros Organics, Geel, Belgium). At such prepared suspension of bacteria, the turbidity was established between 3 and 4 according to McFarland scale, which corresponds to estimated bacteria number of 10.5∙10^8^ cells/mL. This suspension was used for the inoculation of bare and Pt-coated glass slides.

The sterilization of bare and Pt-coated glass slides—necessary before starting biologic examinations—was achieved through placing the substrates in 12-well plates and immersing them for 1 h in 70% ethanol (obtained by dilution of 99.8% ethanol, Acros Organics, Geel, Belgium). This method of sterilization did not change the surface properties of the materials. After this, ethanol was removed, and the samples were rinsed three times with distilled water. Next, they were left to dry.

Consecutively, 0.1 mL of bacterial saline suspension was applied on bare and Pt-coated glass slides. Then, 2 mL of culture nutrient-poor growth medium, containing 10-g/L tryptone (BTL, Warsaw, Poland), 5 g/L yeast extract (BTL, Warsaw, Poland) and 10 g/L physiological saline (Acros Organics, Geel, Belgium) at pH = 7, was added. Culturing of bacteria was carried out for 2 days in an incubator at 35 °C. Samples for analysis were taken after 3 h, 24 h and 48 h.

In order to obtain reliable results, the experiments were performed in triplicate for all materials under the same conditions. The results were presented as mean values ± standard deviation. A Student’s *t*-test was performed to determine the statistical significance (*p* < 0.05).

### 2.3. Staining and Imaging Bacterial Cells Adhered to Glass Slides

The two-color fluorescence assay LIVE/DEAD® BacLight Bacterial Viability Kit (Life Technologies, Thermo Fisher Scientific, Waltham, MA, USA) was used to stain bacteria; live bacterial cells were labeled in green with SYTO9 stain, whereas dead bacterial cells were labeled in red with D-propidium iodide. Confocal fluorescent microscope (Olympus FluoView FV1000, Tokyo, Japan) was used to visualize bacteria on bare (noncoated) and Pt-coated glass slides, and then to analyze the number of live and dead cells. Image analysis was accomplished using ImageJ software (NIH, Bethesda, MD, USA).

Scanning electron microscopy was used to visualize and analyze the morphology of bacteria grown on bare and Pt-coated glass surfaces. Before the samples could be visualized, the materials underwent several stages of conditioning (dehydration). Consequently, the materials were fixed using 3% glutaraldehyde (Fisher BioReagents, Waltham, MA, USA) for 24 h and dehydrated by immersing the samples in the solutions of ethanol (Acros Organics) with increasing concentrations (30%, 50%, 70%, 80%, 90%, 95%, 99.8%), and then dried in the dryer (24 h, 50 °C). Dehydrated samples were sputter-coated with a gold layer for better image quality (20 min, 20 mA; Q150R Quorum Technologies, Lewes, UK), and then their surface was examined by scanning electron microscopy (Phenom ProX, Thermo Fisher Scientific, Waltham, MA, USA). Images were taken with an accelerating voltage of 15 kV at the magnifications of 1000×, 5000× and higher. The average length and width of bacteria, as well as their density (number of bacteria per 200 µm^2^) were calculated using the ImageJ software (NIH, Bethesda, MD, USA).

## 3. Results and Discussion

### 3.1. Materials Characterization

**Figure 1:**
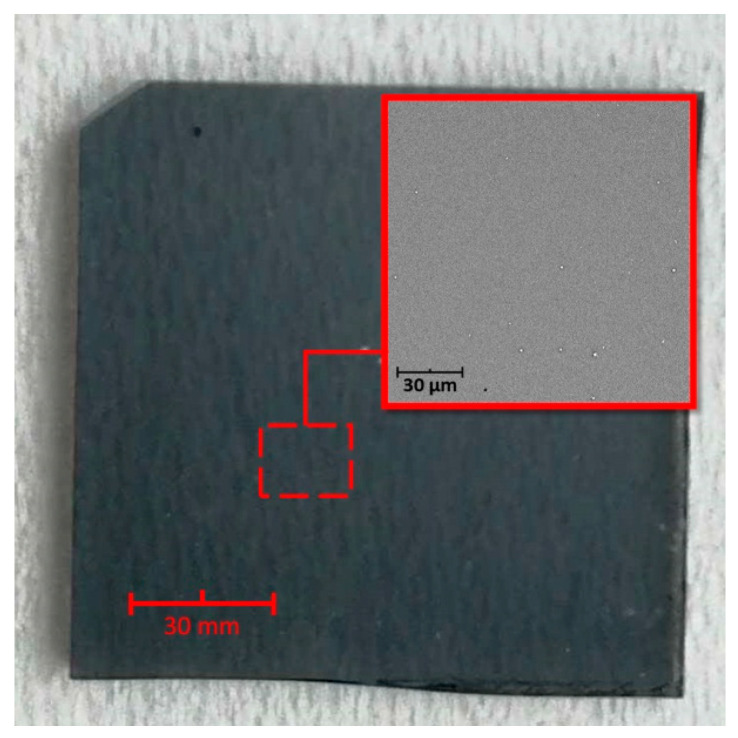
Homogeneity of a sputter-coated layer of platinum. Optical image of a Pt-coated glass slide and a SEM image as the inset.

This article concerns the investigations of antibacterial properties of a thin layer of platinum sputter-coated on the surface of a glass slide in relation to a noncoated slide (bare glass). Sputter coating allows for a full surface coverage, resulting in the formation of a thin coating with a distinct metallic gloss. Imaging of the sputter-coated sample using SEM confirmed that platinum is present on the surface of a glass slide and forms a homogeneous layer (Figure 1). Surface properties, such as surface roughness, charge, wettability or surface energy, are among many material features that can influence the phenomenon of bacterial adherence [25,26]. Literature data indicate that higher roughness [27,28] and more hydrophobic nature of a surface [29,30,31] result in facilitated adhesion of bacteria. However, these effects are observed particularly in the first stages of surface colonization by bacterial cells, and they vanish as the biofilm becomes mature [28,32].

The surface area roughness of bare and Pt-coated glass slides, expressed by the arithmetical mean height (S_a_), varied slightly, retaining values of 0.26 ± 0.01 µm for a bare glass slide and 0.21 ± 0.01 µm for a Pt-coated glass slide, respectively. Even though the difference in S_a_ was slight, it was supposed to affect the adhesion of bacteria. The greater roughness promotes the adhesion, as shown by Han et al. [27], who analyzed the development of *Streptococcus mutans, Streptococcus sanguinis* and polymicrobial (Microcosm) biofilms on modified surfaces of titanium. A significantly higher number of bacteria of each strain was observed on the surface of titanium that was sandblasted with large grits and acid-etched (S_a_ of 1.4 µm), than on the surface of pickled titanium disk (S_a_ of 0.3 µm). However, during biofilm maturation, the effect of surface roughness decreased. Therefore, based on the surface roughness analysis, it was supposed that the attachment of bacteria to the surface of bare glass should be slightly enhanced as compared with Pt-coated glass.

**Figure 2:**
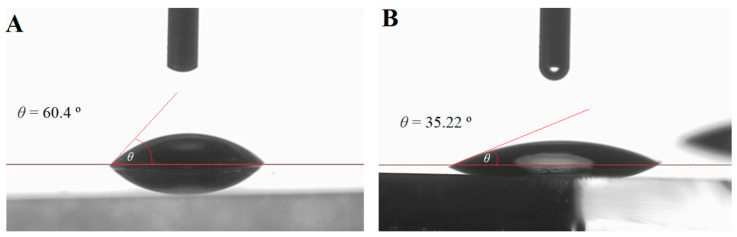
Surface hydrophilicity of investigated surfaces. (**A**) Behavior of a drop of water on the surface of a bare glass slide and (**B**) a Pt-coated glass slide.

Another important surface feature that can significantly affect the process of bacterial adhesion is surface hydrophilicity. It is accepted that bacteria with hydrophilic properties prefer hydrophilic material surfaces, whereas the ones with hydrophobic characteristics prefer hydrophobic surfaces. Consequently, a difference in the wettability between bare glass slides and Pt-coated slides was investigated by determining the contact angle (θ) at room temperature (T ≈ 20 °C) using deionized water. The results (Figure 2) showed that the surface of Pt-coated glass was evidently more hydrophilic (θ = 35.2° ± 10.5°) than a surface of bare glass (θ = 60.4° ± 7.5°). According to the literature data [32,33,34,35], the adhesion effect of various hydrophilic bacteria, including *Escherichia coli*, is usually enhanced on hydrophilic surfaces, but at a different extent. Bacterial cells show a higher affinity to surfaces with moderate wettability as compared with extremely hydrophobic or hydrophilic surfaces [34]. Thus, it was expected that *Escherichia coli* should exhibit a higher affinity towards Pt-coated glass than to bare one.

As the potential effects of surfaces roughness and hydrophilicity on the attachment of bacteria were ambiguous—and a layer of platinum could lead to the decrease (basing on surface roughness data) or to the increase (basing on its hydrophilic character) in bacterial adhesion—there was a need to carry out biologic investigations on the behavior of a selected bacterial strain colonizing both types of surfaces.

### 3.2. Bacterial Colonization of Materials Surface

**Figure 3:**
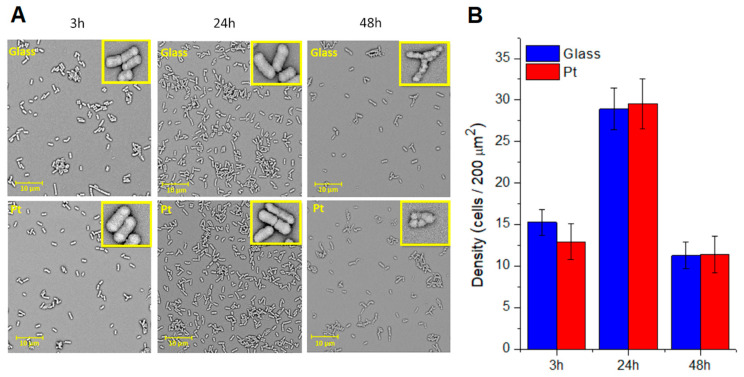
Colonization of *Escherichia coli* on the surfaces of bare and Pt-coated glass slides. (**A**) SEM micrographs presenting bacterial populations grown for 3 h, 24 h and 48 h on the surface of samples; (**B**) bacterial cells density (number of bacterial cells per 200 µm^2^) calculated from SEM images.

To assess the ability of bacteria to attach and grow on bare and Pt-coated glass slides, *Escherichia coli*, as a common pathogen, was cultured on the surface of materials for 48 h. After specified time, samples were investigated using SEM, and the micrographs were used to visualize the interactions between bacteria and investigated surfaces (Figure 3A). Consecutive stages of bacterial growth were monitored by assessing the bacterial cell density after 3 h, 24 h and 48 h of incubation. Accordingly, the results obtained after 3 h of culturing were related to the initial phase of bacteria growth, in which the bacteria tendency to attach to the surface could be investigated. As observed in Figure 3B, the density of bacteria on bare glass (15.3 ± 1.6 cells/200 µm^2^) was slightly higher than on a Pt-coated glass slide (12.9 ± 2.1 cells/200 µm^2^), what was consistent with the literature data [27,32], indicating a better adhesion of bacteria to more rough surface, although obtained data don’t show statistically significant differences. These results may also suggest that the effect of roughness dominates over the effect of hydrophilicity, making the surface of a Pt-coated glass slide less prone to bacterial attachment.

Comparing to the first stage of bacterial growth (3 h of culture), where bacteria were irreversibly bound to the surface, the next phase (24 h) represented the multiplication stage of bacteria that occurred after their attachment to the surface. At this time, the bacterial cells multiplied and started spreading over the surface of specimens. In this phase, significantly increased number of bacteria was observed on both types of surfaces, namely 28.9 ± 2.5 cells/200 µm^2^ and 29.5 ± 3.0 cells/200 µm^2^ for bare and Pt-coated glass slides, respectively (Figure 3B). Since the bacteria density on both types of surfaces was almost identical, it could be concluded that the initial inconsiderable decrease in the rate of adhesion observed on the surface of Pt was only a short-term effect, overcame by the ability of bacteria to proliferate.

After 48 h, a significant decrease in bacterial density on both surfaces was evident. This time point indicated a death phase of bacterial growth since the bacteria did not receive a new portion of fresh culture medium during this experiment. Therefore, many bacterial cells observed in the micrographs possessed deformed shapes (Figure 3A—insets). From the distorted surface of bacterial cells, it could be concluded that the growth and reproduction of bacteria were disrupted. Due to this, dead bacteria underwent lysis, released cells debris, and were easily detached from the surface. For this reason, only few healthy cells were observed on the surface of samples, and the bacterial density reached the values even lower than in the initial phase, i.e., 11.3 ± 1.6 cells/200 µm^2^ and 11.4 ± 2.3 cells/200 µm^2^ for a bare glass and a Pt-coated glass slide, respectively. These identical results pointed to the high similarity of both surfaces in terms of material characteristics influencing the adhesion of bacteria.

### 3.3. Dimensions of Bacterial Cells

*Escherichia coli* has already been extensively analyzed, becoming one of the best-known models of Gram-negative and relatively anaerobic bacteria. *Escherichia coli* is a rod-shaped bacterium with the average cell length of 1–2 µm and width of 0.5–1.0 µm. These dimensions depend on the bacterial metabolism and intensity of growth, since *Escherichia coli* can easily modulate its size to maintain vitality and reproductive capacity depending on the availability of nutrient-rich medium [36,37].

**Figure 4:**
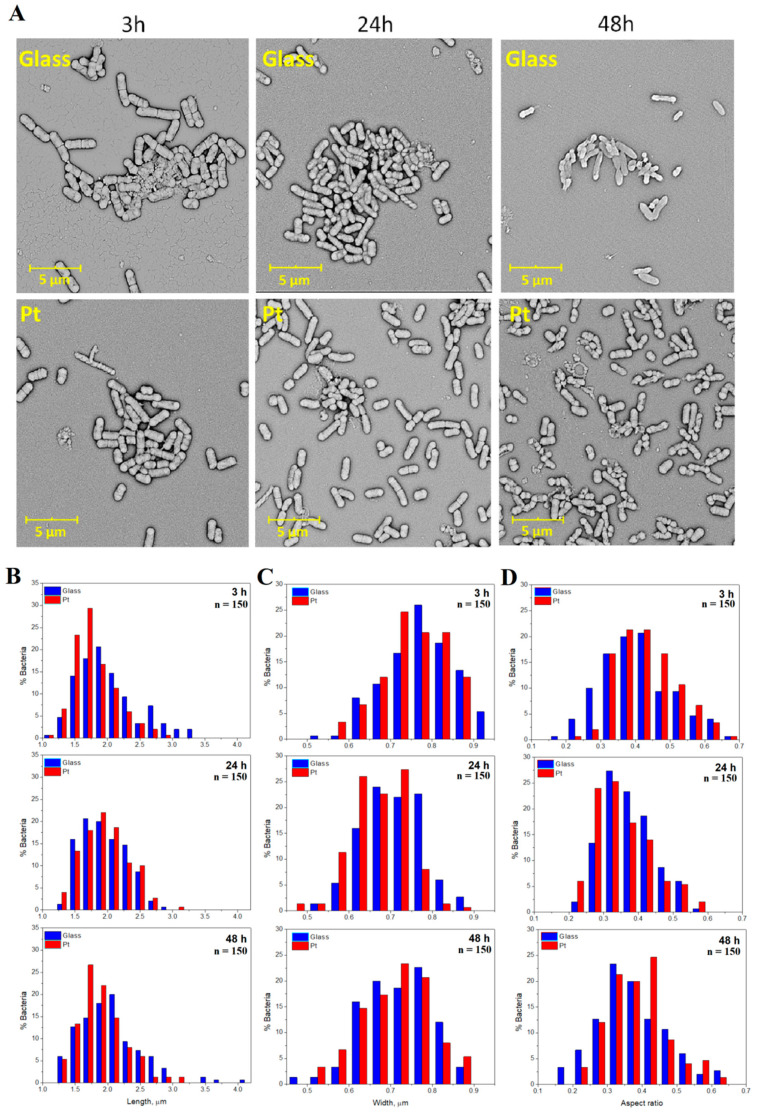
Size distribution of bacterial cells on bare and Pt-coated glass slides. (**A**) SEM micrographs presenting bacterial populations grown for 3 h, 24 h and 48 h on bare and Pt-coated glass; (**B**) histograms of the bacterial cells percentage in terms of specified values of (**C**) length, width and (**D**) aspect ratio, calculated from SEM images; n = 150.

Therefore, to analyze the growth of bacteria on the surfaces of both materials (Figure 4A), their dimensions (length and width) were assessed (Table 1). To numerically describe the shape of the bacteria independently of their sizes, the dimensionless aspect ratio was introduced and calculated as a function of width and length. The values of the aspect ratio approached zero for very elongated bacteria and were close to unity for circular cells. In general, the investigated bacteria possessed average dimensions of rod-shaped *Escherichia coli* cells, namely the average length between 1.79 µm and 2.04 µm, and the average width between 0.66 µm and 0.77 µm. Although having similar shape, the bacteria cells cultured on the surface of bare glass were longer and wider (length of 1.95–2.04 µm, width of 0.70–0.77 µm) than the cells grown on the surface of Pt-coated glass (length of 1.79–1.95 µm, width of 0.66–0.75 µm).

The analysis of histograms showing the variations in length, width and aspect ratio (Figure 4B–D) was performed to provide more information and to assess the cycle of bacterial proliferation. Changes in the average length of bacterial cells, observed in Figure 4B after 3 h of incubation on both bare glass and Pt-coated glass, suggested that the cells remained still in the lag phase. After 24 h, bacteria cultured on glass were longer than in case of bacteria cultured on Pt, suggesting that their metabolic activity was enhanced. In addition, the bacteria cultured on glass were thicker than bacteria cultured on Pt (Figure 4C), confirming preferential growth of bacteria on glass. Furthermore, the bacteria were the widest after the first 3 h of incubation, while in subsequent time points (24 h and 48 h) they were longer in order to divide into daughter cells. After 24 h, all bacteria were found to grow actively (an increase in length was noted), which allowed them to enter the division phase and to significantly increase their population. A significant increase in length of bacterial cells was observed on the Pt-coated glass, for which the average value exceeded the result observed on bare glass.

However, after 48 h the length of cells was found to decrease. This effect, together with a significant decrease in cell density (Figure 3), suggested that bacteria entered the death phase. At this stage, the higher number of shorter cells was observed on bare glass as compared with the Pt-coated glass. However, the opposite effect was observed for cells being 1.4–2.0 mm long. In addition, the distribution of cells with lengths in the range of 1.4–2.2 mm (comprising lengths characteristic for *Escherichia coli*) was similar to that noted in the first phase. Some bacterial cells occupying the surface of bare glass were considerably longer than bacteria on Pt-coated glass. Comparing the aspect ratio of cells (Figure 4D), more frequently lower values were observed for bacteria growing for 24 h on both bare and Pt-coated glass, indicating intensive cell proliferation at that time.

### 3.4. LIVE/DEAD Analysis

**Figure 5:**
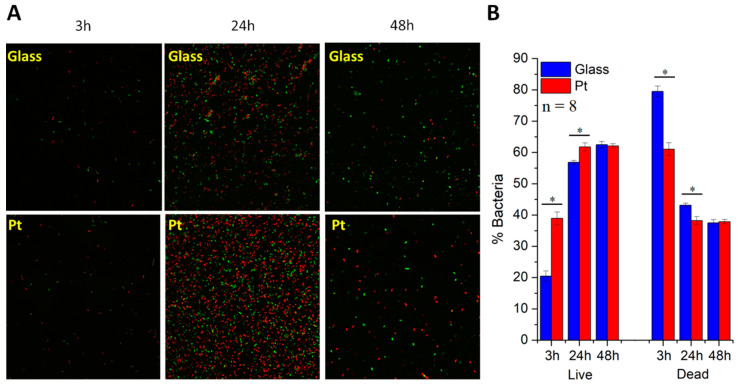
Viability of *Escherichia coli* on surfaces of glass and Pt-coated glass slides. (**A**) Confocal fluorescent microscopic images and (**B**) percentage values of live and dead bacteria after 3 h, 24 h and 48 h; * = *p* < 0.05, n = 8.

To assess bacterial viability, a LIVE/DEAD analysis based on confocal fluorescent microscopic images was carried out at each time point (Figure 5A). The set of data (Figure 5B) indicated that, despite the higher density of bacteria on the surface of bare glass after first 3 h of culture, a significant part of them (79.5% ± 1.7%) was dead. The percentage of dead bacteria was significantly lower (61.1% ± 2.0%) during bacteria growth on Pt-coated glass. This result showed that a thin film of Pt deposited on a surface did not exhibit any antibacterial effects but seemed to be more compatible towards bacteria than a glass control surface. The percentage of live bacteria was twice as high on the surface of a Pt-coated glass than a glass control (38.9% ± 2.0% and 20.5% ± 1.7%, respectively).

This tendency partially vanished at the subsequent time point (after 24 h of incubation) representing the growth phase of bacterial culture. Now, live bacteria that adhered to the surface during the initial phase started to multiply, leading to a significant increase in cell density. After 24 h of incubation, the increased percentage of live bacteria on both types of surfaces was also observed. This effect was more pronounced in the case of Pt-coated glass than bare glass (61.8% ± 1.3% and 56.8% ± 0.6%, respectively).

Despite the fact that there were only a few bacteria remaining on the slides after 48 h, most of them were alive. The number of live bacteria was almost identical for both bare and Pt-coated glass slides (62.5% ± 1.1% and 62.1% ± 0.7%, respectively). On platinum, the percentage of live bacteria remained similar as in the previous time point. The 3D reconstruction of bacteria cultured on the surface of a Pt-coated glass slide for 48 h (Supplementary file) indicated that some cells were attached perpendicularly to the investigated surface, which should be associated with hydrophilicity of the substrate [38].

Since previous studies reported the antibacterial effect of sputter-coated layers of copper and silver [17,18], it was expected that also sputter-coated layer of platinum should possess similar biocidal character. This hypothesis was supported by the fact that both Pt ions and Pt nanoparticles are known for their antibacterial activity. Pt ions, due to their electronegativity, are supposed to be highly attracted to the negatively charged bacteria [2]. As a consequence, the increase in the bacterial-metal ion interactions is observed, leading to cell death. Small Pt nanoparticles, on the other way, are supposed to easily pass through the pores of the bacterial membrane, affecting cell growth and viability [14]. We expected similar results for a sputtered layer of Pt since the sputter-coating process should produce a platinum film with the grain size of about 2 nm. However, our results revealed opposite observations to those expected, as a thin layer of sputtered Pt was not shown to exhibit any antibacterial effect.

The lack of antibacterial properties of sputter-coated Pt layer should be correlated with the physicochemical stability of deposited film, preventing Pt ions and nanoparticles from release. In contrast, the mechanism of antimicrobial activity of sputter-coated silver and copper films was based on the release of Ag or Cu, respectively [17]. In the case of a mixed layer of Ag–Cu, the antibacterial effect could also arise from the galvanic action and small electrical field generated by the Ag–Cu electrochemical couple, as suggested by Blenkinsopp et al. [39]. For a thin film made purely of Pt, electric field could not be generated without external stimulation.

Thus, high physicochemical stability of platinum being its great advantage is also responsible for the lack of biocidal character of a sputter-coated layer against a model Gram-negative bacterial strain, *Escherichia coli*. Therefore, the surface of thin Pt film may be easily colonized by bacterial population, leading to an increased risk of infections, including nosocomial infections. For this reason, there is an urgent need for the development of new methods of surface modification that will reduce the risk of its colonization by bacteria and subsequent infections.

## 4. Conclusions

In this study, bare glass and Pt-coated glass slides were used to investigate the antibacterial activity of sputter-coated Pt films against a model bacterial strain, *Escherichia coli*. It was shown that the interactions between bacteria and both materials were similar. Although the examined materials were found to differ in hydrophilic character, roughness had a stronger effect on the adhesion of bacteria than wettability. Therefore, the surface of a Pt-coated glass slide was less prone to bacterial attachment than a bare glass slide. Unfortunately, the lower number of bacterial cells adhering to the Pt-coated glass observed initially was only a short-term effect. The density of bacteria on both types of surfaces increased considerably after 24 h of culture. Consequently, it was shown that a thin layer of sputtered Pt was not sufficient to cause any antibacterial effects.

The lack of expected biocidal character of sputter-coated Pt layer against *Escherichia coli* seems to be correlated with the high physicochemical stability of such thin film, preventing Pt ions and Pt nanoparticles from release. Therefore, even though platinum is a metal exhibiting high biocompatibility and superior electrochemical properties and has a great importance and potential in biomedical applications, this study shows its serious limitation in the context of colonization by bacterial population, particularly *Escherichia coli*. For this reason, there is an urgent need for the development of new methods of surface modification that will reduce the risk of surface colonization by bacteria and subsequent infections, but will maintain high physicochemical stability and electroactivity of Pt.

## Figures and Tables

**Table 1:** Average dimensions of bacterial cells. Comparison of average values of length, width and aspect ratio of bacteria cultured on bare and Pt-coated glass slides for 3 h, 24 h and 48 h; * = *p* < 0.05, n = 150.

Surface	Glass			Platinum		
**Time, h**	3	24	48	3	24	48
**Length, µm**	2.00 ± 0.04 *	1.94 ± 0.03	2.04 ± 0.04 *	1.79 ± 0.03 *	1.95 ± 0.03	1.90 ± 0.03 *
**Width, µm**	0.77 ± 0.01	0.70 ± 0.01 *	0.71 ± 0.01	0.75 ± 0.01	0.66 ± 0.01 *	0.71 ± 0.01
**Aspect ratio**	0.40 ± 0.01 *	0.37 ± 0.01	0.37 ± 0.01	0.43 ± 0.01 *	0.35 ± 0.01	0.39 ± 0.01

## Supplementary Materials

The following are available online at https://www.mdpi.com/1996-1944/13/12/2674/s1, Video S1: E.coli-on-sputter-coated-Pt-film_48h.
